# A case of co-occuring synesthesia, autism, prodigious talent and strong structural brain connectivity

**DOI:** 10.1186/s12888-020-02722-w

**Published:** 2020-06-30

**Authors:** Andreas Riedel, Simon Maier, Kerstin Wenzler, Bernd Feige, Ludger Tebartz van Elst, Sven Bölte, Janina Neufeld

**Affiliations:** 1 Section for Experimental Neuropsychiatry, Department for Psychiatry & Psychotherapy, Medical Center – University of Freiburg, Faculty of Medicine - University of Freiburg, Freiburg, Germany; 2 Center of Neurodevelopmental Disorders (KIND), Centre for Psychiatry Research; Department of Women’s and Children’s Health, Karolinska Institutet, Stockholm Health Care Services, Region Stockholm, Stockholm, Sweden; 3 Curtin Autism Research Group, Essential Partner Autism CRC, School of Occupational Therapy, Social Work and Speech Pathology, Curtin University, Perth, Western Australia; 4 Child and Adolescent Psychiatry, Stockholm Health Care Services, Region Stockholm, Stockholm, Sweden

**Keywords:** Synesthesia, Autism, Prodigious talent, Brain connectivity, Language

## Abstract

**Background:**

Synesthesia is a sensory phenomenon where certain domain-specific stimuli trigger additional sensations of e.g. color or texture. The condition occurs in about 4% of the general population, but is overrepresented in individuals with Autism Spectrum Disorder (ASD), where it might also be associated with the presence of prodigious talents.

**Case presentation:**

Here we describe the case of a young transsexual man with Asperger Syndrome, synesthesia and a prodigious talent for foreign language acquisition. In our case, not only letters, numbers, spoken words, music, noises, weekdays and months lead to highly consistent, vivid color sensations but also his own and others’ emotions, geometric shapes, any mathematical symbol, and letters from an unfamiliar alphabet (Hebrew). These color associations seem to aid categorization, differentiation and storage of information and might thereby contribute to the young man’s language acquisition ability. We investigated the young man’s structural brain connectivity in comparison to adults with or without ASD, applying global fiber tracking to diffusion-weighted Magnetic Resonance Imaging (MRI) data. The case presented with increased connectivity, especially between regions involved in visual and emotion processing, memory, and higher order associative binding regions. An electroencephalography experiment investigating synesthetic color and shape sensations while listening to music showed a negligible occipital alpha suppression, indicating that these internally generated synesthetic sensations derive from a different brain mechanism than when processing external visual information.

**Conclusions:**

Taken together, this case study endorses the notion of a link between synesthesia, prodigious talent and autism, adding to the currently still sparse literature in this field. It provides new insights into the possible manifestations of synesthesia in individuals with ASD and its potential contribution to prodigious talents in people with an otherwise unexceptional cognitive profile. Additionally, this case impressively illustrates how synesthesia can be a key element not only of sensory perception but also social and emotional processing and contributes to existing evidence of increased brain connectivity in association with synesthesia.

## Background

Synesthesia is a sensory condition where certain sensory stimuli lead to additional, internally generated (concurrent) sensations within the same or a different sensory modality. One of the most common synesthesia types is sequence-color synesthesia where linguistic sequences such as letters, numbers, or weekdays induce sensations of color [1, 2]. Four main characteristics define developmental (in contrast to acquired or pharmacologically induced) synesthesia [3]. First, presence since childhood. Second, consistency of the synesthetic sensations; for instance, the color evoked by a certain letter should always be the same. Third, the vividness (sensory-like quality) of the synesthetic sensations and fourth, their automatic and involuntary occurrence. It is gold standard to verify sequence-color synesthesia using an objective test [4], where synethetes, in contrast to non-synesthetes, typically report highly consistent colors for each inducing stimulus, e.g. always mint green for the letter “N”, even if re-assessed after years [2]. A computerized version of this consistency test [5] reliably differentiates synesthetes from non-synesthetes [6].

The prevalence of synesthesia has been estimated to be about 4% in the general population [7]. While synesthesia runs in families and seems to be at least partly under genetic control [8–10] it is genetically heterogeneous [11–13]. Synesthetic color experiences are commonly associated with activity within sensory brain regions together with parietal and frontal cortex areas known to be crucial for feature integration [2]. These synesthetic brain activation patterns are likely the result of altered brain connectivity [14, 15]. Increased brain connectivity has been observed in synesthetes, for instance between parietal and sensory regions [16–18], but also globally [19, 20]. However, connectivity alterations depend on individual differences between synesthetes, such as the location of the synesthetic color experiences, which can be perceived as if they were projected on the inducing stimulus (“projector synesthetes”) vs on a mental screen (“associator synesthetes”). More specifically, projector synesthetes might use a more bottom-up mechanism within the temporal lobe, while associator synesthetes seem to recruit a more top-down mechanism via parietal or frontal brain regions [18, 21]. In line with these findings, reduced stimulus-related parieto-occipital alpha-suppression in response to synesthesia inducing letters in projector synesthetes as compared to both associators and non-synesthetes might indicate reduced parietal top-down influences in this sub-group [22]. In addition to individual differences, methodological limitations likely contributed to the between-study variability regarding the brain regions associated with synesthesia [23].

Interestingly, synesthesia is more common in individuals with Autism Spectrum Disorder (ASD) [24–26]. ASD is a neurodevelopmental condition with a prevalence of 1–2.6% [27, 28], characterized by impairing patterns of social communication and interaction alongside restricted/repetitive patterns of behavior, interests, or activities [29]. It is further commonly associated with altered sensory processing [30]. Like synesthesia, ASD is a complex polygenetic condition [31] with a large phenotypic variety and associated with inconsistent and wide-spread alterations in brain connectivity. One recent structural MRI multi-center study investigating a large sample of individuals with and without ASD, carefully controlling for methodological confounders, found only small group differences in ventricular volume, volume of the central segment of the corpus callosum and altered cortical thickness in occipital regions [32].

A link between synesthesia, ASD and prodigious talent was already proposed in 2007, by Simon Baron-Cohen and colleagues who described the case of Daniel Tammet, a man with Asperger syndrome who also experiences synesthesia and possesses prodigious talents, similar to those commonly observed in people with “savant syndrome” [33]. Daniel can make fast, complicated calculations in his mind and has an extensive memory for numbers, enabling him to cite the number pi for 22,514 decimal places after the comma. Since the likelihood of developing all three conditions (synesthesia, ASD, and Savant syndrome) independently is very low, Baron-Cohen and colleagues argued that these conditions are linked and that the co-occurrence of autism and synesthesia might increase the likelihood to possess prodigious talents. This is in line with preliminary evidence that synesthesia is specifically more common in individuals with ASD who additionally report prodigious talents [26] and another case with ASD, savant syndrome and synesthesia [34]. Synesthesia-like mapping between different ‘systems’ (e.g. colors, letters, numbers, days of the week, musical notes etc.) might play an important role in the development of prodigious talents [26, 34–36]. Concurrent synesthetic experiences might facilitate memory for, or discrimination of, inducing stimuli by enriching them and enhancing the attention towards them [33, 35]. In line with this view, synesthetes perform better in certain memory and sensory discrimination tasks [37–39]. Moreover, synesthetes show alterations in sensory processing, similar to people with ASD, including altered sensory sensitivity and a more detail-oriented visual attention [40–42]. Hence, alterations in general sensory information processing might play a role in the etiology of both conditions.

In the current study, we describe a young transsexual man, LP, diagnosed with Asperger syndrome, who came to our attention because he described multiple types of synesthesia very vividly when assessed for ASD. In contrast to previously described cases, he experiences visual synesthetic sensations (color, shape/texture) for a large proportion of his day-to-day sensory input (visual, auditory, gustatory, olfactory), internal states (emotions), and even stimuli that are new to him (unknown symbols or letters). He further possesses an exceptional talent for learning new languages, which enabled him to learn to understand and speak Spanish in only a few days. Here, we describe LP’s synesthesia, his synesthetic consistency, his autistic symptoms and his cognitive profile. Further, we compared his structural brain connectivity to neurotypical (NT, here defined as not diagnosed with ASD or any other neurodevelopmental condition) controls and adults with ASD diagnosis, using a hypothesis-free global tracking approach which has not been utilized to investigate synesthesia previously. Finally, we used EEG in order to examine his occipital alpha suppression response to music inducing synesthesia as a possible sign of low-level visual system involvement.

## Case presentation

### Biographical information

LP was born in Northern Germany in 1998 as the younger of two children of a Russian mother and a German father. When he was in the first class of school, LP mentioned the colors that numbers induce in him to his mother – who was not surprised since she experienced colors for letters and numbers herself. LP grew up as biologically female. Already in preschool, he noticed that he was different from the other children and felt like belonging to neither sex. He had difficulties to make friends, to put himself into other’s shoes and to understand irony and metaphors. In school, LP did well in most subjects but suffered from being bullied by other children. Like many individuals with ASD who are (biologically) of female sex, he received his ASD diagnosis rather late, here at the age of 18. Around the same time, LP also decided to take the steps to change gender. He started taking male hormones in March 2017, changed his name shortly after and a mastectomy is planned. When coming to the follow-up analysis in September 2018 at the age of 20 years, LP had started studying physics and made friends. He also reported having found several new strategies, helping him to cope with situations that used to be difficult for him, including both sensory over-stimulation and social challenges.

### Diagnostic information

LP was diagnosed with Asperger Syndrome in 2016 in a clinical department in northern Germany and his diagnosis was confirmed at the University Medical Center Freiburg in July 2017. There was no indication for other psychiatric or neurological conditions such as psychosis, depression, epilepsy or migraine nor delayed language acquisition. Consensus diagnosis was confirmed by a team of experts, following the recommendations of the National Institute for Health and Clinical Excellence for Autism in Adults and the German S3-Criteria. LP completed the Autism Diagnostic Observation Schedule-2 (ADOS-2), where he exceeded the cut-off for ASD in the communication subscale. However, he scored below the cut-off in the social interaction subscale and the combined score’s cut-off.

LP’s mother completed the “Marburger Beurteilungsskala zum Asperger Syndrom” (MBAS) and the German version of the “Australian Scale for Asperger’s Syndrome” (ASAS) [43] in 2015 and the parent-report Social Responsiveness Scale (SRS) [44] in 2018. In the ASAS, LP scored 120 out of 144 points in total. In the MBAS, LP exceeded cutoff for the total score and the sub-scales assessing theory of mind difficulties (sub-scale score = 46), stereotyped and contextually inappropriate behaviors (sub-scale score = 33) and alterations in articulation and motor behavior (sub-scale score = 36) but not in shared joy, attention, facial expressions and gestures (sub-scale score = 19). LP’s total raw score on the SRS was 79, which is within the autistic range.

### LP’s synesthesia

As long as he can remember, LP experiences colors for almost every kind of linguistic stimulus, including letters, numbers, and other symbols (e.g. mathematical symbols or geometric shapes), weekdays and months. Further, he experiences color and often also shapes and textures for tastes, smells, all kinds of sound including music, his own feelings and emotions and other people’s characters and their emotions. He even experiences colors for stimuli that are completely new for him such as unfamiliar alphabets, unfamiliar (mathematical) symbols or shapes. LP reported that the color of letters depends for him on both the visual shape and the sound/pronunciation. This becomes especially clear when comparing the colors of letters from different alphabets. For instance, in the Cyrillic alphabet, “H” is pronounced like the English/German “N” and LP sees it in light green, which is mix of his white German “H” and the green German “N”. For new written stimuli, the color is mostly related to the shape and can change slightly once he knows the symbol’s meaning or the letter’s pronunciation. For some stimuli, he also experiences a shape, consistency, or texture, which then is also associated with the color. For instance, the letter “R” for him is the only black letter “because it is the hardest one” and the letter “H” is white “because it is the softest letter”. Additionally, LP has several types of *Spatial Sequence Synesthesia*, where the elements of different sequences (days of the week, months of the year, numbers, the alphabet, his playlist of music titles) form together a shape in space. For instance, his music playlist is like a landscape of hills and valleys – and when browsing through his playlist it feels for him like he is moving on this landscape in his mind.

### Objective assessment of LP’s synesthesia

LP completed the online Synesthesia Battery (https://www.synestheste.org; see Supplement) in summer 2018, where the consistency of his color choices for Latin letters and digits 0–9 and four further alphabets (Greek, Cyrillic, Czech and Hebrew) was assessed using objective tests [5]. Additionally, he completed color-consistency tests for months, weekdays, chords and musical instruments. LP scored way below even a more conservative cutoff of 1.0 in all these tests (Latin alphabet and digits: 0.37, Cyrillic alphabet: 0.53, Greek alphabet: 0.37, Czech alphabet: 0.4, and Hebrew alphabet: 0.56, months: 0.35, weekdays: 0.37, chords: 0.88, musical instruments: 0.45), indicating highly consistent synesthetic colors (higher scores indicate more inconsistent / variable color choices). Hebrew was not familiar to him, but since he experiences color automatically even for unfamiliar symbols he indicated experiencing synesthesia for this alphabet and was tested for it. In an additional test (Speeded Congruency Test) the automaticity of a participant’s color experiences is assessed. For this, the Latin letters and digits are presented in synesthetically congruent (=color the participant chose in the previous test) or incongruent color. Participants indicate via button press (left and right mouse button) as fast as possible whether the color is congruent or incongruent with the perceived synesthesia. In the Speeded Consistency test, LP responded at 95.85% accuracy with a mean reaction time of 1.10 s (+/− 0.56).

### Projector vs associator status

Within the synesthesia battery, LP also completed a questionnaire designed to differentiate projector and associatior synesthetes [45]. Scores between − 4 and + 4 are possible and positive scores indicate projector status while negative score indicate associator status. LP scored 3.35, which identifies him as a clear projector synesthete for letters/numbers. This matches his verbal descriptions for these and other synesthesia types he experiences. For instance, LP sees colors on and in other people’ bodies in association with their personality and their emotional expressions.

### Talents

We interviewed LP about prodigious talents, giving him a list of examples of such talents as used in the study of Hughes et al. [26]. We explained to LP that a prodigious talent is an ability that only very few people have. LP reported to have certain strength in language and memory. As an example, he named that when he started learning Spanish at the age of 16, he could already after 3 days understand 95% of the words from newspaper articles and news and engage in conversations on several day-to-day topics. After that, LP continued studying Spanish auto-didactically at a lower intensity but on a regular basis for at least half a year. We asked LP without warning to complete an online test (https://www.lengalia.com) on his Spanish level, where he reached level C1 of the Common European Framework of Reference for Languages. This is the fifth and next highest level (speaking fluent Spanish in a complex manner; https://www.coe.int/en/web/common-european-framework-reference-languages) and is usually reached after several years of training. Further, besides German and Russian he also speaks English and Polish fluently, reported to be able to understand Latin, and speak French, Dutch, Swedish and Catalan at a basic level. LP reported to have learned Spanish in an inductive (bottom-up) fashion by extracting meaning from songs, texts and TV shows based on the sentence structure and already known words. Whenever gaining new knowledge this way, LP experiences a *blue aura* of euphoria, a sensation of a shiny blue with green reflections like the neck of a peacock. In contrast to most other languages, Spanish induced a strong *blue aura* in him, which was an important factor motivating him to learn this language.

### Cognitive profile

LP’s general cognitive ability was assessed with the Wechsler Intelligence Scale for Adults, 4th version (WAIS-IV). LP scored exactly one standard deviation above the population mean in three sub-tests of the WAIS, namely Matrix Reasoning, Digit Span, Digit-Symbol Coding, which depend strongly on acoustic and visual working memory, concentration and abstract thinking. In the remaining sub-tests he scored within one standard deviation above or below the general population mean. In line with his relative strength in the digit span, LP mentioned that it is easy for him to learn lists of numbers and he learned more than 100 decimals of number pi on 1 day several years ago. When we asked him without warning to cite pi as far as he could remember, he could still correctly cite it until the 97th digit after the comma.

### Brain imaging assessment

MRI assessment was performed on a 3 T Siemens (Erlangen, Germany) PRISMA Magnetom equipped with a 64-channel head coil, including a high-resolution structural scan and a diffusion-weighted scan (see Supplement). Image processing was performed using the in-house software NORA (http://www.nora-imaging.com/). All 61 spatial directions of the diffusion-weighted images were taken into account for the calculation of the diffusion tensor. After co-registration of the structural image onto the Diffusion Tensor Imaging (DTI) image, the global tracking algorithm was applied to simulate the white matter fiber architecture with so-called stream tracks [46]. Based on 56 Gy matter areas defined in the LBPA40 atlas [47], a connectivity matrix was calculated which indicates the weighted number of stream tracks with which each atlas region of interest (ROI) is linked to the other atlas regions (ROI-to-ROI connections).

**Fig. 1:**
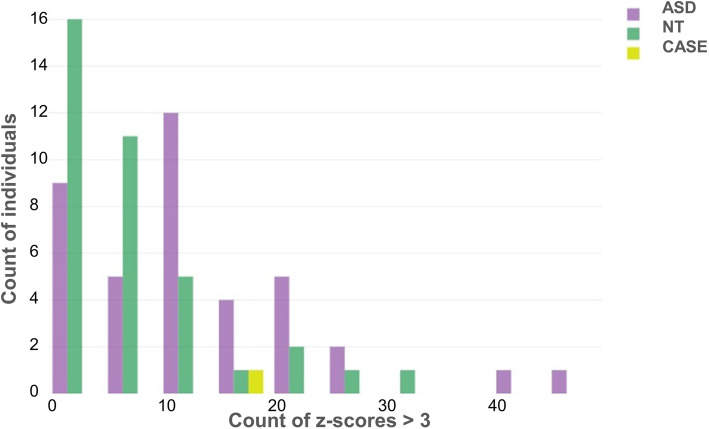
Histogram of number of individuals (y-axes) according to the number of connections with a z-score > 3 (x-axes). LP (in yellow) had 18 connections with z-score > 3, i.e. 18 connections with a higher fiber count compared to the whole sample. Nine participants of the ASD group (purple) and four participants of the NT group (green) had more than 18 such connections while the majority of control participants had less than 18 such connections

**Fig. 2:**
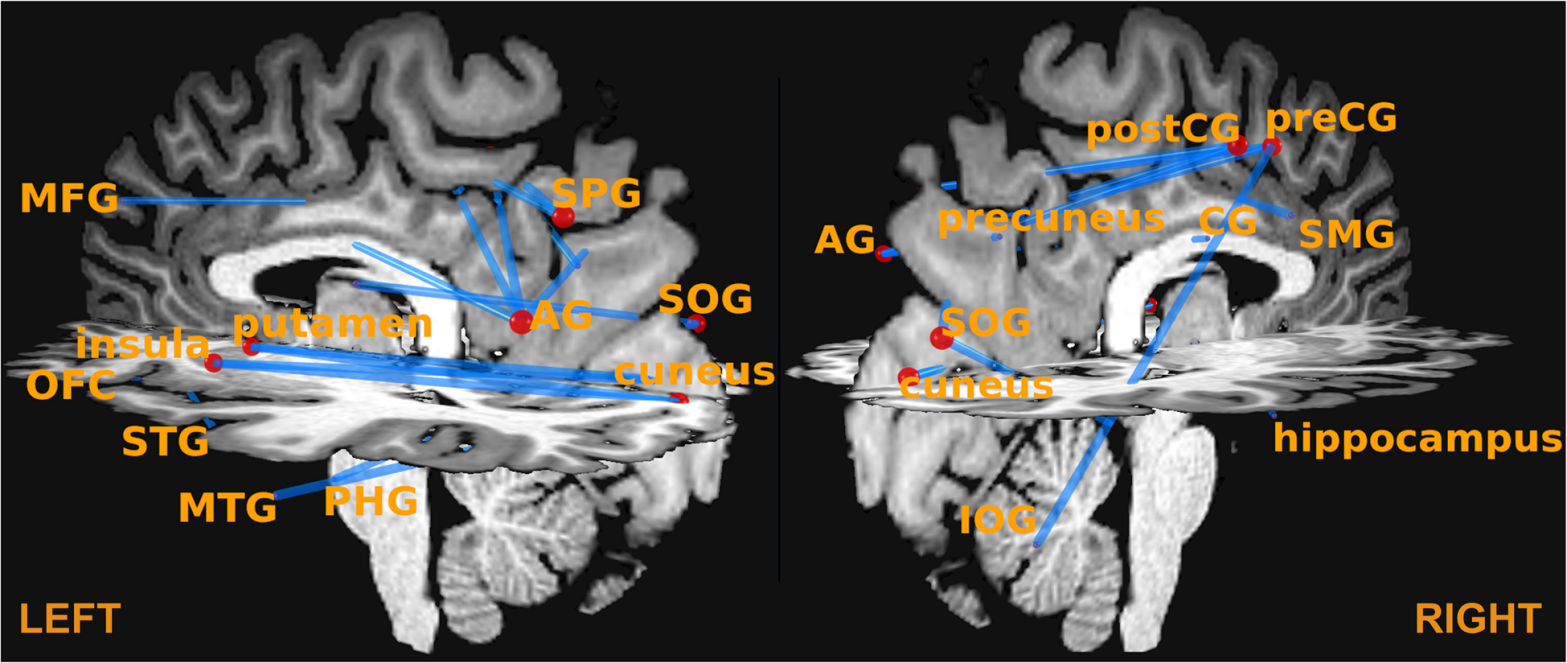
Connections between gray matter areas according to the LBPA40 atlas with a z-score > 3 in LP. The red circles indicate the center of each anatomical region (edges), with larger radius indicating a larger number of connections, and the blue lines indicate the connections (links). AG = angular gyrus; CG = cingulate gyrus; IOG = inferior occipital gyrus; MFG = middle frontal gyrus; MTG = middle temporal gyurs; lat. OFC = lateral orbitofrontal gyrus; PHG = parahippocampal gyrus; postCG = posterior central gyrus; preCG = precentral gyrus; SMG = supramarginal gyrus; SOG = Superior occipital gyrus; SPG = superior parietal gyrus; STG = superior temporal gyrus

**Fig. 3:**
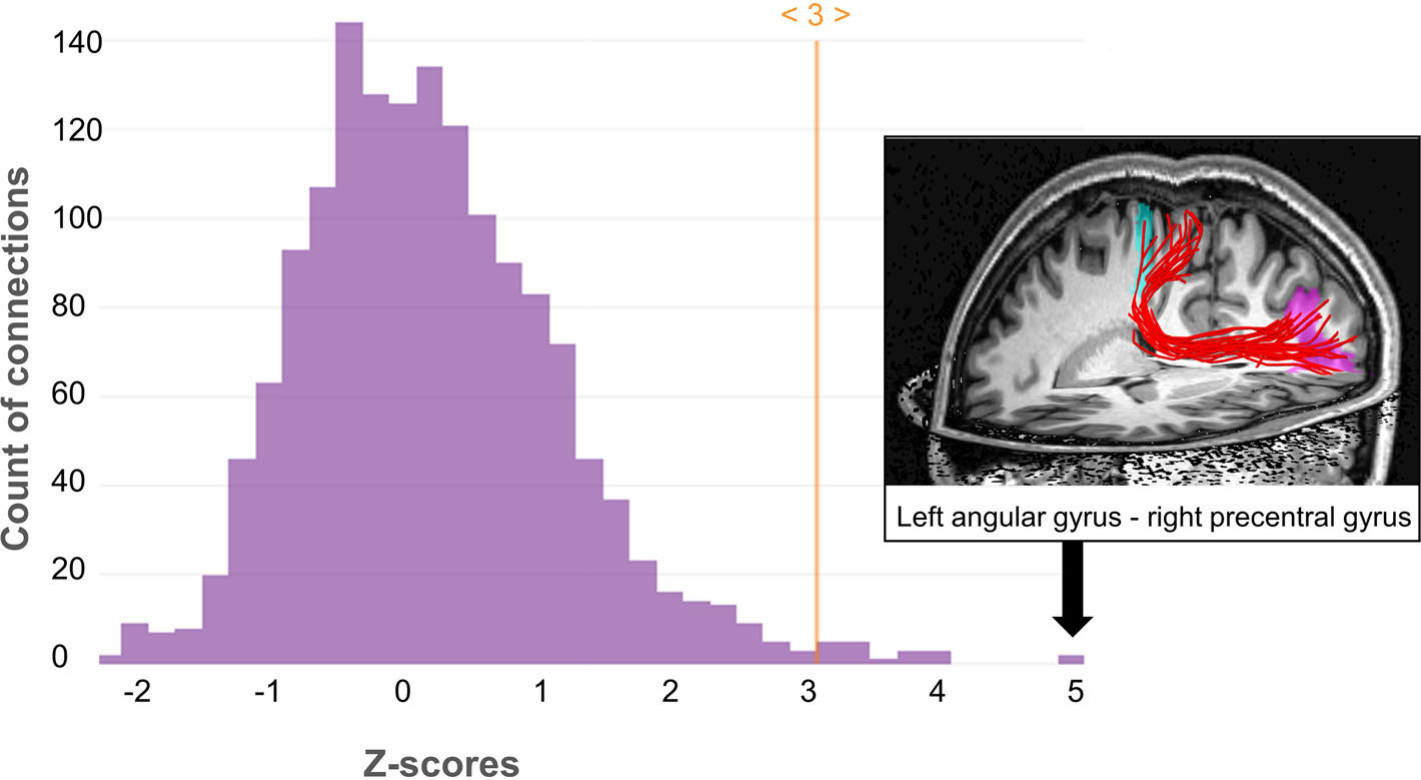
Histogram of the frequency of LP’s z-scores across all LBPA40 atlas regions (compared to all controls). All z-values exceeding the set threshold of 3 were positive, indicating increased connectivity in LP. The most extreme value (> 4.9) was the connection between left angular gyrus (magenta) und right precentral gyrus (cyan), whose fiber trajectory is shown as stream tracks in a front left view of the brain

Two control groups, ASD patients (*N* = 39) and NT controls (*N* = 37), were assessed using identical imaging sequences and image processing procedures (see Supplement). The counts of all ROI-to-ROI connections in each participant’s connectivity matrix was z-transformed across all subjects. To detect “extreme” connectivity patterns, the number of connections with a z-score below − 3 or above 3 (approx. 3 Standard deviations off the mean) was calculated for each subject. This z-value threshold was chosen because it provides a manageable number of possibly unusual connections considering the large number of possible connections (56 regions with 1540 possible connections). The number of such “extreme” connections was then counted per participant. LP had 18 such “extreme” connections, all with a z-score above 3, while the majority of participants from both control groups (78% from the ASD group and 89% from the NT group) had less than 18 such connections (see Figs. 1 & 2). In comparison to the ASD group alone, LP exhibited 12 connections with a z-score > 3, of which 11 connections were the same as found in comparison to the whole group, including several fronto-parietal connections, insula-cuneus connections and the connection between lateral orbitofrontal gyrus and hippocampus (see Table 1). Compared to NT controls alone, additional connections were stronger in LP, especially between frontal cortex regions and fusiform gyrus and between lingual gyrus and pre- and postcentral gyrus. Compared to females (ASD + NT; *N* = 23), LP had stronger connectivity between parietal regions (superior parietal cortex and angular gyrus) to pre- and postcentral gyrus and between precuneus and angular gyrus. Figure 3 shows the frequency of z-scores of all connections of LP as a histogram together with the stream tracks (simulating the fiber trajectories) of LP’s most extreme connection between left angular gyrus and the right pre-central gyrus is in a front left view of LP’s brain.

**Table 1:** Connections between atlas regions that were stronger (3 z-scores above corresponding group mean) in LP compared to different comparison groups

Region one	Region two	LP > ALL	LP > NT	LP > ASD	LP > F
Left middle frontal gyrus	Right supramarginal gyrus	X	X	X	
Right middle frontal gyrus	Left fusiform gyrus		X		
Right inferior frontal gyrus	Right fusiform gyrus		X		
Left precentral gyrus	Right gyrus rectus		X		
	Left lingual gyrus		X		
Right precentral gyrus	Left superior parietal gyrus	X	X	X	X
	Left angular gyrus	X	X	X	X
	Right inferior occipital gyrus	X		X	
	Left lingual gyrus		X		
Left lateral orbitofrontal gyrus	Right hippocampus	X	X	X	
Left gyrus rectus	Left superior temporal gyrus	X	X		
	Left middle temporal gyrus			X	
Right postcentral gyrus	Left superior parietal gyrus	X	X	X	X
	Left angular gyrus	X	X	X	X
	Left precuneus	X			
	Left lingual gyrus		X		
Left supramarginal gyrus	Right superior occipital gyrus	X	X		
Right precuneus	Left angular gyrus	X	X	X	X
Right superior occipital gyrus	Left parahippocampal gyrus	X	X		
Left insular cortex	Left cuneus	X	X	X	
	Right cuneus	X	X	X	
Left cuneus	Left putamen	X			
	Left middle temporal gyrus	X			
Right cingulate gyrus	Left angular gyrus	X	X		
Left putamen	Right cuneus	X		X	

Connections that were stronger (z-score > 3) in LP compared to different comparison groups

*ALL* whole sample, *NT* neurotypical group, *ASD* participants diagnosed with ASD, *F* female control group (12 NT + 11 ASD)

### Electroencephalography

A 64-channel EEG was recorded using a SynAmps 2 amplifier system (Compumedics NeuroScan, Charlotte, USA), low-pass filtered at 100 Hz and stored with a sampling rate of 500 Hz. Eyes were opened (Eyes open condition) and closed (Eyes closed condition) for 30s each, timed by a stimulation program, with five repetitions (total time 5 min). Afterwards, LP listened to self-selected music with closed eyes for 5 min (Music condition). Effects of horizontal and vertical eye movements on the EEG were corrected using Independent Component Analysis (ICA). Spectral analysis was performed on artifact-free segments of 1024 data points (2.048 s, spectral resolution 0.49 Hz), logarithmized and averaged.

**Fig. 4:**
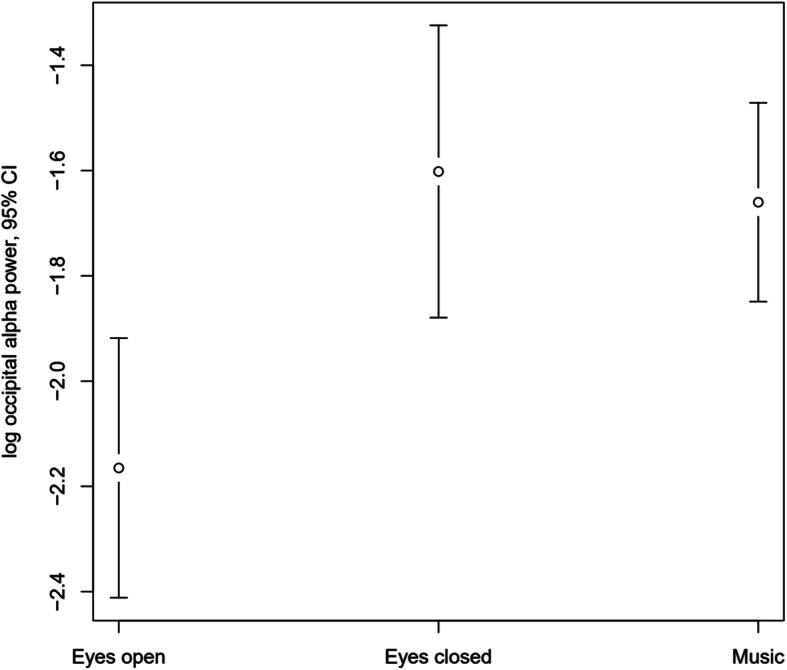
Logarithmic occipital EEG alpha power in the three conditions (Eyes open, Eyes closed and Music). Error bars represent 95% confidence intervals (CI)

An occipital alpha peak was present in the eyes closed condition at 11 Hz and clearly reduced (though not absent) in the eyes open condition (alpha suppression; *p* = 0.002). The incomplete alpha suppression may indicate drowsiness. Alpha power during the Music condition was practically indistinguishable from the Eyes closed condition (*p* = 0.637, see Fig. 4).

## Conclusions

### Discussion

Here we present the case of LP, a young, transsexual man diagnosed with Asperger syndrome, who experiences multiple types of synesthesia and seems to have an extraordinary talent for learning languages.

LP’s results from the synesthesia battery test confirm the consistency and automaticity of his color sensations. As he also reported to experience the synesthetic colors vividly and as long as he can remember, his synesthesia fulfills all criteria of developmental synesthesia.

Since almost all kinds linguistic (letters, numbers, symbols, words, weekdays, months, etc.) and sensory (sound/music, taste, smell) stimuli lead to vivid visual sensations of color and shape or texture for LP, his everyday perception is strongly enriched and dominated by synesthetic sensations – a private world which he strongly enjoys. This mapping of external input to his personal color system might thereby enable a more organized categorization of sensory input, which has been suggested to contribute to the development of prodigious talents [26, 34, 36]. For instance, people with sequence-space synesthesia have an advantage in learning ‘calendar calculation’ based on a set of rules after intensive training [48]. Baron-Cohen et al. [33] proposed that a plausible reason for the co-occurrence of savant skills and synesthesia might be due to a facilitation effect of synesthesia on memory. This might be because the concurrent synesthetic experiences enrich the percept, making it thereby more memorable or easier to discriminate from similar stimuli. This view is supported by better memory performance in synesthetes for stimuli related to their synesthetic experiences, but additionally there is also evidence for increased memory in domains unrelated to synesthetic sensations, even though these results are more mixed [37]. LP described that he uses synesthetic colors when trying to memorize words, remembering for instance that the word was green first before he remembers the word itself. LP’s synesthesia might also have enhanced his ability to distinguish different words and pronunciations. For instance, he described to perceive distinct visual patterns for a word when pronounced correctly as compared to when pronounced with a slight accent, making small differences more distinguishable for LP – something most people learning a new language often struggle with. Synesthesia also influences the joy and motivation associated with language learning for LP via the pleasant and interesting synesthetic sensations accompanied with the new words. He described how the Spanish language led to strong sensation of the *blue aura*, which for him was a strong motivation factor to learn this language, while he had little motivation to learn other languages where he experienced the *blue aura* rarely or never (e.g. Dutch). On the other hand, LP also performed in the higher normative range on standard memory tests in the current study, without any exceptional cognitive peaks, suggesting that his memory advantage might be either rather subtle or restricted to specific tasks like learning languages.

LP had stronger ROI-to-ROI connections than 89% of the NT sample and 78% of the ASD sample. However, a few participants from the control samples had even more extreme (strong or weak) connections than LP. Many of the connections that were found to be unusually strong in LP in comparison to both control groups and the ASD group alone, were those of brain regions involved in lower-level vision, including both lower-level (occipital) and higher level (parietal) visual areas. In LP, we also observed increased connectivity of the right hippocampus, a core region for memory encoding and retrieval. Treffert [49] argues that the prodigious talents in people with savant syndrome always depend on prodigious memory and hence LP increased hippocampal connectivity might be linked to his potential prodigious language talent. Consistent with this view, altered structural connectivity (right hemispheric increase and left-hemispheric decrease) of core brain structures of memory functioning including the hippocampus have been reported in a previous case with savant syndrome [50].

LP also showed increased brain connectivity of the insula, where altered gray matter structure has been reported in projector synesthetes in comparison to controls [21]. He also showed increased connectivity of regions previously indicated in associator synesthetes (precentral gyrus, precuneus and middle frontal gyrus) [21]. Our most stable results (observed in comparison to all control groups: NT, ASD, and females only) were increased connectivity of the left superior parietal gyrus / left angular gyrus with the right precentral and postcentral gyrus and left angular gyrus with right precuneus. This corresponds to increased white matter integrity (indicated by fractional anisotropy, FA) in the left parietal cortex found in both projector and associator synesthetes compared to controls [45]. Parietal brain regions have earlier been suggested to trigger synesthetic experience by projecting back to sensory regions that represent the synesthetic concurrent sensations [15–17, 51]. Especially the angular gyrus has been suggested to be involved in methaphor processing and synesthetic sensations [52]. The precuneus might also be involved in LP’s synesthesia since it is involved in various integration functions including episodic memory retrieval, gestalt integration and mental imagery [53]. Consistent with this view, the precuneus has been implicated in several synesthesia types previously, namely lexical–gustatory, lexical-color, grapheme-color and people color synesthesia [54–57]. In addition, it is also a core region of the default mode network, which has been suggested to be involved in a general monitoring and evaluation of external and internal sensory information when no (task-) directed attention is required [58]. Further, LP showed increased occipital connectivity of the superior occipital gyrus compared to all controls, overlapping with Brodmann area 19, where increased functional connectivity has previously been found in association with synesthetic color experience induced by letters [17]. On the other hand, we did not find increased connectivity of the inferior temporal cortex (including color area V4) or the superior frontal cortex which have been indicated to have stronger structural connectivity in synesthetes [45].

Taken together, some of LP’s connectivity alterations correspond to regions that have previously been indicated in synesthesia and might hence be part of the mechanism underlying LP’s synesthetic perception while other regions, such as the hippocampus, might be involved in LP’s language talent.

In the EEG experiment, LP showed practically the same mean occipital alpha amplitude for both, the Eyes closed and the Music condition. We can conclude that LP’s synesthetic visual experience happens without concomitant occipital alpha reduction in the eyes-closed state, indicating that LP’s synesthetic visual experience in response to music is not based on the same brain mechanisms as processing external visual stimuli. In contrast, a previous study found reduced stimulus-related parieto-occipital alpha-suppression in response to synesthesia inducing letters in projector synesthetes as compared to both associators and non-synesthetes [22]. However, this previous study differed from our study in several ways, which can explain the contradicting results. While we compared 30s long stimulus conditions in line with common approaches to assess state alpha, Cohen et al. [22] examined event-related spectral changes. Further, they compared groups of synesthetes to controls, targeted grapheme-color synesthesia rather than music synesthesia, had both inducing and non-inducing stimuli and participants performed a task during the assessment.

### Strengths & Limitations

In this case study, we present a case with Asperger syndrome and an unusually rich presentation of synesthesia, which we describe in detail based on the patient’s (LP) report. We further assessed all types of synesthesia where this was possible with an objective test, confirming that they were highly consistent and hence genuine. We also present a detailed summary of the patient’s symptoms and cognitive profile. Further, we investigated his structural brain connectivity in comparison to several control groups, using a hypothesis-free approach. However, the generalizability of findings in single cases is naturally limited. In order to identify distinctive structural connectivity patterns in LP, we screened for z-values of LP that exceeded 3 or − 3. Unfortunately, a case study does not allow any further statistical tests to calculate the probability of such extreme test results, given that the null hypothesis is correct (no difference between groups). Accordingly, no procedures for the correction (of the *p*-value) of multiple comparisons could be applied. Hence, little can be concluded regarding the reliability and generalizability of the reported results. Although we had both, an NT and an autistic control group in the DTI assessment, we have no possibility to decipher alterations in brain connectivity related to LP’s synesthesia, his prodigious talent, or other individual differences. Since we did not assess LP’s functional brain connectivity during synesthetic processing or language acquisition, we also have no direct evidence that the observed structural connectivity findings were indeed related to synesthesia or memory functions. Moreover, we did not assess any control group in the EEG experiment and hence we can only conclude that LP’s alpha suppression with closed eyes was not significantly modulated by his synesthetic response to music, but not that his alpha suppression was within the range of neurotypical alpha suppression. Finally, while we did test LP’s Spanish level with an objective test, we had no possibility to measure the amount of practice LP needed to reach this level and hence our conclusion that LP learned this language unusually fast is solely based on his own report.

### Final conclusions

We present a unique case with a remarkable rich synesthesia phenotype, Asperger Syndrome and an exceptional strength in language learning. In order to get an unbiased picture of this case’s structural brain connectivity in comparison to other individuals with ASD and NT controls, we used global tracking approach. Our case’s brain imaging results correspond to previous reports of increased brain connectivity in synesthetes, especially in parietal brain regions. Further, the case LP shows that despite an otherwise unexceptional cognitive profile, synesthesia may foster specific talents – such as the effortless, intuitive and inductive acquisition of new languages. Additionally, the case of LP impressively demonstrates how synesthesia can be a key element for emotional experience, perception and ‘being-in-the-world’.

## Supplementary information

**Additional file 1.**

## Data Availability

The datasets generated and analyzed during the current study are not publicly available due to data protection reasons, but are available from the corresponding author on reasonable request under the condition that LP explicitly consents to this.

## Abbreviations

ASD: Autism Spectrum Disorder
DTI: Diffusion Tensor Imaging
EEG: Electroencephalography
ICA: Independent Component Analysis
MRI: Magnetic resonance imaging
MPRAGE: Magnetization-prepared rapid gradient echo
NT: Neurotypical
ROI: Region of interest
WAIS-IV: Wechsler Intelligence Scale for Adults, 4th version

## References

[1] Gray BF, Simner J. Synesthesia and release phenomena in sensory and motor grounding. Cases of disinhibited embodiment? Front Psychol. 2015;6:54. 10.3389/fpsyg.2015.00054 PMC4311609 25688227

[2] Ward J (2013). Synesthesia. Annu Rev Psychol.

[3] Sinke C, Halpern JH, Zedler M, Neufeld J, Emrich HM, Passie T (2012). Genuine and drug-induced synesthesia: a comparison. Conscious Cogn.

[4] Baron-Cohen S, Wyke MA, Binnie C (1987). Hearing words and seeing colours: an experimental investigation of a case of synaesthesia. Perception..

[5] Eagleman DM, Kagan AD, Nelson SS, Sagaram D, Sarma AK (2007). A standardized test battery for the study of synesthesia. J Neurosci Methods.

[6] Rothen N, Seth AK, Witzel C, Ward J (2013). Diagnosing synaesthesia with online colour pickers: maximising sensitivity and specificity. J Neurosci Methods.

[7] Simner J, Mulvenna C, Sagiv N, Tsakanikos E, Witherby SA, Fraser C (2006). Synaesthesia: the prevalence of atypical cross-modal experiences. Perception..

[8] Barnett KJ, Finucane C, Asher JE, Bargary G, Corvin AP, Newell FN (2008). Familial patterns and the origins of individual differences in synaesthesia. Cognition..

[9] Baron-Cohen S, Burt L, Smith-Laittan F, Harrison J, Bolton P (1996). Synaesthesia: prevalence and familiality. Perception..

[10] Bosley HG, Eagleman DM (2015). Synesthesia in twins: incomplete concordance in monozygotes suggests extragenic factors. Behav Brain Res.

[11] Asher JE, Lamb JA, Brocklebank D, Cazier J-B, Maestrini E, Addis L (2009). A whole-genome scan and fine-mapping linkage study of auditory-visual synesthesia reveals evidence of linkage to chromosomes 2q24, 5q33, 6p12, and 12p12. Am J Hum Genet.

[12] Tomson SN, Avidan N, Lee K, Sarma AK, Tushe R, Milewicz DM (2011). The genetics of colored sequence synesthesia: suggestive evidence of linkage to 16q and genetic heterogeneity for the condition. Behav Brain Res.

[13] Tilot A, Kucera KS, Vino A, Asher J, Baron-Cohen S, Fischer S (2018). Rare variants in axonogenesis genes connect three families with sound–color synesthesia. PNAS..

[14] Grossenbacher PG, Lovelace CT (2001). Mechanisms of synesthesia: cognitive and physiological constraints. Trends Cogn Sci.

[15] Rouw R, Scholte HS, Colizoli O (2011). Brain areas involved in synaesthesia: a review. J Neuropsychol.

[16] Neufeld J, Sinke C, Zedler M, Dillo W, Emrich HM, Bleich S (2012). Disinhibited feedback as a cause of synesthesia: evidence from a functional connectivity study on auditory-visual synesthetes. Neuropsychologia..

[17] Sinke C, Neufeld J, Emrich HM, Dillo W, Bleich S, Zedler M (2012). Inside a synesthete’s head: a functional connectivity analysis with grapheme-color synesthetes. Neuropsychologia..

[18] Van Leeuwen TM, Den Ouden HE, Hagoort P (2011). Effective connectivity determines the nature of subjective experience in grapheme-color synesthesia. J Neurosci.

[19] Dovern A, Fink GR, Fromme ACB, Wohlschläger AM, Weiss PH, Riedl V (2012). Intrinsic network connectivity reflects consistency of synesthetic experiences. J Neurosci.

[20] Hänggi J, Wotruba D, Jäncke L (2011). Globally altered structural brain network topology in grapheme-color synesthesia. J Neurosci.

[21] Rouw R, Scholte HS (2010). Neural basis of individual differences in synesthetic experiences. J Neurosci.

[22] Cohen MX, Weidacker K, Tankink J, Scholte HS, Rouw R (2015). Grapheme-color synesthesia subtypes: stable individual differences reflected in posterior alpha-band oscillations. Cogn Neurosci.

[23] Hupé J-M, Dojat M (2015). A critical review of the neuroimaging literature on synesthesia. Front Hum Neurosci.

[24] Baron-Cohen S, Johnson D, Asher J, Wheelwright S, Fisher SE, Gregersen PK (2013). Is synaesthesia more common in autism?. Mol Autism.

[25] Neufeld J, Roy M, Zapf A, Sinke C, Emrich HM, Prox-Vagedes V (2013). Is synesthesia more common in patients with Asperger syndrome?. Front Hum Neurosci.

[26] Hughes JE, Simner J, Baron-Cohen S, Treffert DA, Ward J (2017). Is synaesthesia more prevalent in autism spectrum conditions? Only where there is prodigious talent. Multisens Res.

[27] Baio J, Wiggins L, Christensen DL, Maenner MJ, Daniels J, Warren Z (2018). Prevalence of autism spectrum disorder among children aged 8 years - autism and developmental disabilities monitoring network, 11 sites, United States, 2014. Morb Mortal Wkly Rep Surveill Summ Wash DC 2002.

[28] Lundström S, Reichenberg A, Anckarsäter H, Lichtenstein P, Gillberg C (2015). Autism phenotype versus registered diagnosis in Swedish children: prevalence trends over 10 years in general population samples. BMJ..

[29] American Psychiatric Association. Diagnostic and statistical manual of mental disorders, (DSM-5®). Arlington: American Psychiatric Pub; 2013.

[30] Hazen EP, Stornelli JL, O’Rourke JA, Koesterer K, McDougle CJ (2014). Sensory symptoms in autism spectrum disorders. Harv Rev Psychiatry.

[31] Talkowski ME, Minikel EV, Gusella JF (2014). Autism spectrum disorder genetics: diverse genes with diverse clinical outcomes. Harv Rev Psychiatry.

[32] Haar S, Berman S, Behrmann M, Dinstein I (2014). Anatomical abnormalities in autism?. Cereb Cortex.

[33] Baron-Cohen S, Bor D, Billington J, Asher J, Wheelwright S, Ashwin C (2007). Savant memory in a man with colour form-number synaesthesia and asperger. J Conscious Stud.

[34] Bouvet L, Donnadieu S, Valdois S, Caron C, Dawson M, Mottron L (2014). Veridical mapping in savant abilities, absolute pitch, and synesthesia: an autism case study. Front Psychol.

[35] Baron-Cohen S, Ashwin E, Ashwin C, Tavassoli T, Chakrabarti B (2009). Talent in autism: hyper-systemizing, hyper-attention to detail and sensory hypersensitivity. Philos Trans R Soc B Biol Sci.

[36] Mottron L, Bouvet L, Bonnel A, Samson F, Burack JA, Dawson M (2013). Veridical mapping in the development of exceptional autistic abilities. Neurosci Biobehav Rev.

[37] Ward J, Field AP, Chin T (2019). A meta-analysis of memory ability in synaesthesia. Memory..

[38] Banissy MJ, Walsh V, Ward J (2009). Enhanced sensory perception in synaesthesia. Exp Brain Res.

[39] Banissy MJ, Tester V, Muggleton NG, Janik AB, Davenport A, Franklin A (2013). Synesthesia for color is linked to improved color perception but reduced motion perception. Psychol Sci.

[40] Ward J, Brown P, Sherwood J, Simner J (2017). An autistic-like profile of attention and perception in synaesthesia. Cortex..

[41] Ward J, Hoadley C, Hughes JE, Smith P, Allison C, Baron-Cohen S (2017). Atypical sensory sensitivity as a shared feature between synaesthesia and autism. Sci Rep.

[42] Van Leeuwen TM, Van Petersen E, Burghoorn F, Dingemanse M, Van Lier R (2019). Autistic traits in synaesthesia: atypical sensory sensitivity and enhanced perception of details. Philos Trans Biol Sci.

[43] Robinson J, Volkmar FR (2013). Australian scale for Asperger’s syndrome. Encyclopedia of autism spectrum disorders.

[44] Constantino JN, Gruber CP (2005). Social responsiveness scale (SRS).

[45] Rouw R, Scholte HS (2007). Increased structural connectivity in grapheme-color synesthesia. Nat Neurosci.

[46] Reisert M, Mader I, Anastasopoulos C, Weigel M, Schnell S, Kiselev V (2011). Global fiber reconstruction becomes practical. Neuroimage..

[47] Shattuck DW, Mirza M, Adisetiyo V, Hojatkashani C, Salamon G, Narr KL (2008). Construction of a 3D probabilistic atlas of human cortical structures. NeuroImage..

[48] Hughes JEA, Gruffydd E, Simner J, Ward J (2019). Synaesthetes show advantages in savant skill acquisition: training calendar calculation in sequence-space synaesthesia. Cortex..

[49] Treffert DA (2009). The savant syndrome: an extraordinary condition. A synopsis: past, present, future. Philos Trans R Soc B Biol Sci.

[50] Corrigan NM, Richards TL, Treffert DA, Dager SR (2012). Toward a better understanding of the savant brain. Compr Psychiatry.

[51] Weiss PH, Zilles K, Fink GR (2005). When visual perception causes feeling: enhanced cross-modal processing in grapheme-color synesthesia. NeuroImage..

[52] Ramachandran VS, Hubbard EM (2003). The phenomenology of synaesthesia. J Conscious Stud.

[53] Cavanna AE, Trimble MR (2006). The precuneus: a review of its functional anatomy and behavioural correlates. Brain J Neurol.

[54] Jones CL, Gray MA, Minati L, Simner J, Critchley HD, Ward J (2011). The neural basis of illusory gustatory sensations: two rare cases of lexical–gustatory synaesthesia. J Neuropsychol.

[55] Nunn JA, Gregory LJ, Brammer M, Williams SC, Parslow DM, Morgan MJ, Morris RG, Bullmore ET, Baron-Cohen S, Gray JA (2002). Functional magnetic resonance imaging of synesthesia: activation of V4/V8 by spoken words. Nat Neurosci.

[56] Steven MS, Hansen PC, Blakemore C (2006). Activation of color-selective areas of the visual cortex in a blind synesthete. Cortex..

[57] Weiss PH, Shah NJ, Toni I, Zilles K, Fink GR (2001). Associating colours with people: a case of chromatic-lexical synaesthesia. Cortex..

[58] Raichle ME, MacLeod AM, Snyder AZ, Powers WJ, Gusnard DA, Shulman GL (2001). A default mode of brain function. Proc Natl Acad Sci.

