# Supplementary material for: A case of co-occuring synesthesia, autism, prodigious talent and strong structural brain connectivity
Source: BMC Psychiatry. 2020 Jun 30;20:342. doi: 10.1186/s12888-020-02722-w (PMC7329514; doi:10.1186/s12888-020-02722-w)
Supplement: Supplementary file 1 — Additional file 1. [file 12888_2020_2722_MOESM1_ESM.docx]

***Supplement***

*Synesthesia Consistency Test*

In the beginning of Synesthesia Battery (https://www.synesthete.org), participants select from a list of known synesthesia types those types they believe to have (1). Subsequently, all indicated synesthesia types for which a test is available are assessed. In each test, stimuli such as letters, weekdays, or musical notes are presented one at a time and the participant selects a color from a color palette of 16.7 million different colors (1) and the smaller the mean difference between the multiple assessments, the higher the consistency. Each stimulus is presented three times, randomly intermixed with the other stimuli in order to prevent participants to simply remember their color choices. For each stimulus, the distance in RGB (= Red, Green, Blue) color space is calculated as mean differences between R, G, and B values between the three trials. The test score is calculated as the mean of all items’ RGB distance. Lower scores indicate more consistent color choices and a cutoff of 1.0 has been suggested as upper border for genuine synesthetes originally (1). Later, a more lenient threshold of 1.43 was found to differentiate more reliably between synesthetes and non-synesthetes (2). In an additional test (Speeded Congruency Test, (1)) the automaticity of a participant’s color experiences for graphemes is assessed. For this, the Latin letters and digits are either presented in the congruent color (the color the participant chose for them in the initial test) or in an inconsistent color that is distant in RGB space from the chosen color. Participants indicate via button press (left and right mouse button) as fast as possible whether the inducer is presented in the consistent or inconsistent color. Similarly as for graphemes, the color consistency of weekday, months, chords and musical instruments is tested within the synesthesia battery. The weekdays and month are presented as written words, the chords (played on a piano) and the instruments (playing all the same short melody) are presented acoustically and could be re-played as often as desired within each trial. At https://www.synesthete.org, researchers have the possibility invite participants to complete the battery online and participants can agree to share all the data their data with the researcher, which is the approach we used for this study.

*Brain Imaging Details*

*Image acquisition and preprocessing.* The MRI session included a standard MPRAGE (magnetization-prepared rapid gradient echo) T1-weighted anatomical scan (relaxation time = 2300ms, echo time = 2.98ms, flip angle = 9°, field of view = 240×256x176mm3, voxel size = 1×1×1mm3) and a diffusion-weighted sequence (relaxation time = 6600ms, echo time = 80ms, flip angle = 90°, field of view = 192×192x120mm3, voxel size = 2×2×2mm3, 61 spatial directions, B-value controlling the diffusion 1000 s/mm2). All EPI volumes were corrected by a rigid body transformation and a distortion correction algorithm for head movement. An experienced clinical neuroradiologist inspected LP’s T1-weighted structural image and concluded that he did not have any structural brain anomalies.

*Control participants.* Two control groups, ASD patients and NT controls, were assessed with approval by the local ethics committee as part of a different study with identical brain imaging sequences as assessed in LP on the same MRI scanner. Participants with insufficient data quality (visible artefacts) or comorbidities were excluded from the analysis. The patients were recruited at the Freiburg Center for the Diagnosis and Treatment of Autism (University center for autism spectrum, German: Universitäres Zentrum Autismus Spektrum Freiburg, UZASF) and the Department for Child and Adolescent Psychiatry Freiburg. The diagnosis was performed by experienced psychiatrists and psychologists according to the guidelines of the National Institute for Health and Clinical Excellence (NICE) for autism in adults and patients who met the diagnostic criteria of ICD-10 for Asperger autism (F84.5) and DSM-5 for ASD (299.00) were included. The ASD control group consisted of 39 adults (28 male), mean age 35.4 (SD=12.0), IQ 119.7 (SD=14.8). NT participants were recruited via announcements in local media and postings on bulletin boards at the University of Freiburg. The NT group consisted of 37 adults (25 male) mean age 34.1 (SD=9.9), IQ 117.9 (SD=12.7). We included both male and female control participants since LP is biologically female but of male gender. Since LP underwent female brain development until age 18, we also compared his brain connectivity to a purely female control group (12 NT, 11 ASD).

*Illustrations of LP’s synesthetic experiences*

The color blue has a special meaning for LP, in particular a kind of blue that he describes as like the blue of the head and neck of the peacock; smooth and shiny but not identical everywhere, with occasional glimpses of green. He described the meaning of this special blue with the neologism “secret-full-ness” (German: “Geheimnissvollheit”). He experiences this special blue within himself and on the person or object associated with a strong feeling of enthusiasm. This happens especially if someone, for instance a character in a TV show, says something very smart or explains a scientific fact to another character. Other emotions or affectively loaded words, but also languages lead to less intense but still very vivid color sensations (**Supplementary Fig.1**). LP also sees people, not only the emotions related to them, in colors covering their entire body, while he sees their actual physical body at the same time. When remembering people and situations with them however, LP mostly sees the synesthetic colors rather than the person’s actual physical form. The colors and structures associated with a person and additional colors triggered by emotions occur at the same time. For instance, LP’s best friend’s basic color is brown and beige with a rather smooth, partly slightly rough structure. When LP sees his best friend laughing, he sees his friend’s face turning bright yellow while the rest of the body remains in the basic colors.

LP learned the German (Latin) alphabet first and the Cyrillic alphabet second. The synesthetic color experienced for Cyrillic letters are similar to according Latin letters if both shape and pronunciation is similar. Otherwise, colors linked to both shape and pronunciation are mixed into a new color (see **Supplementary Fig. 2**).


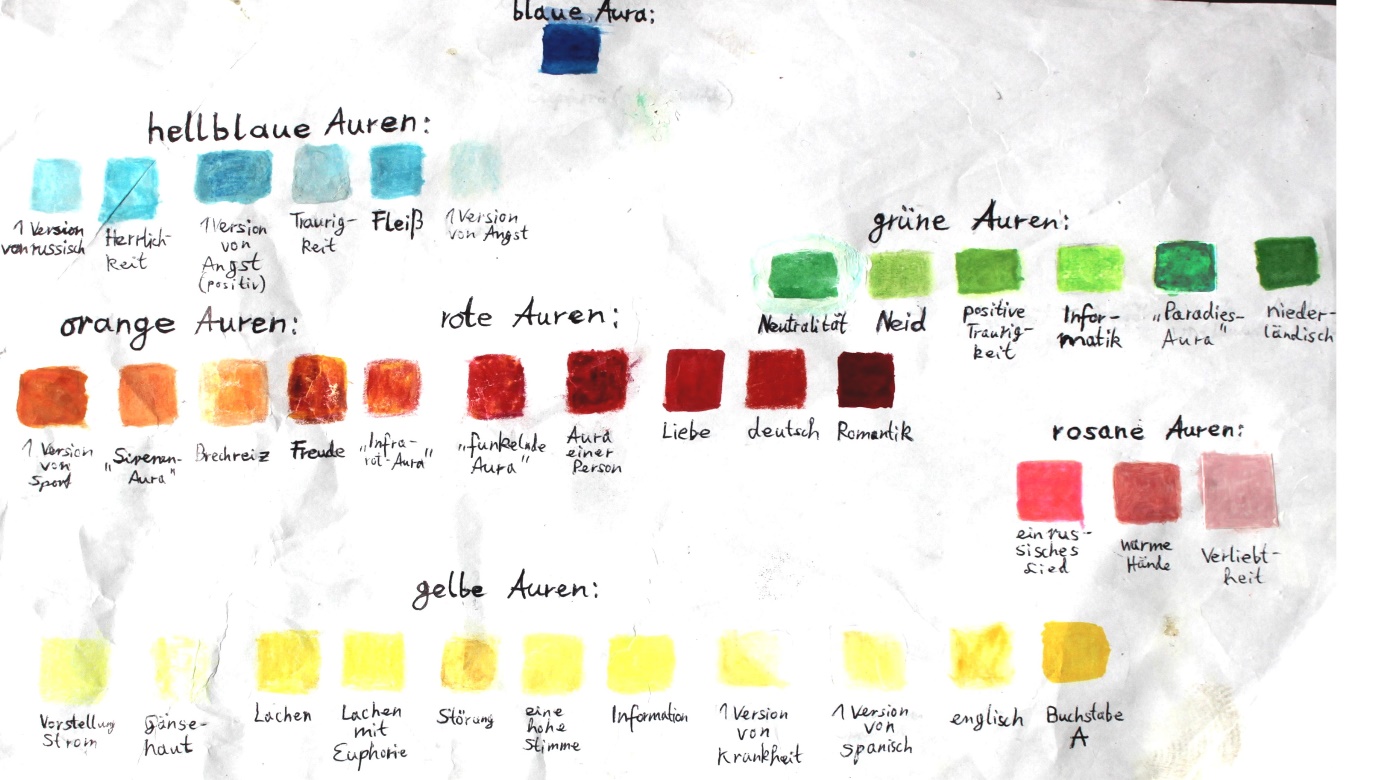


***Supplementary Fig. 1****: Color code for different emotions, affective words or languages sorted by color category. In each category, there are both positive, neutral and negative inducers. For instance in the green category, there is (from left to right in German) neutrality, envy, positive sadness, informatics, paradise and Dutch.*


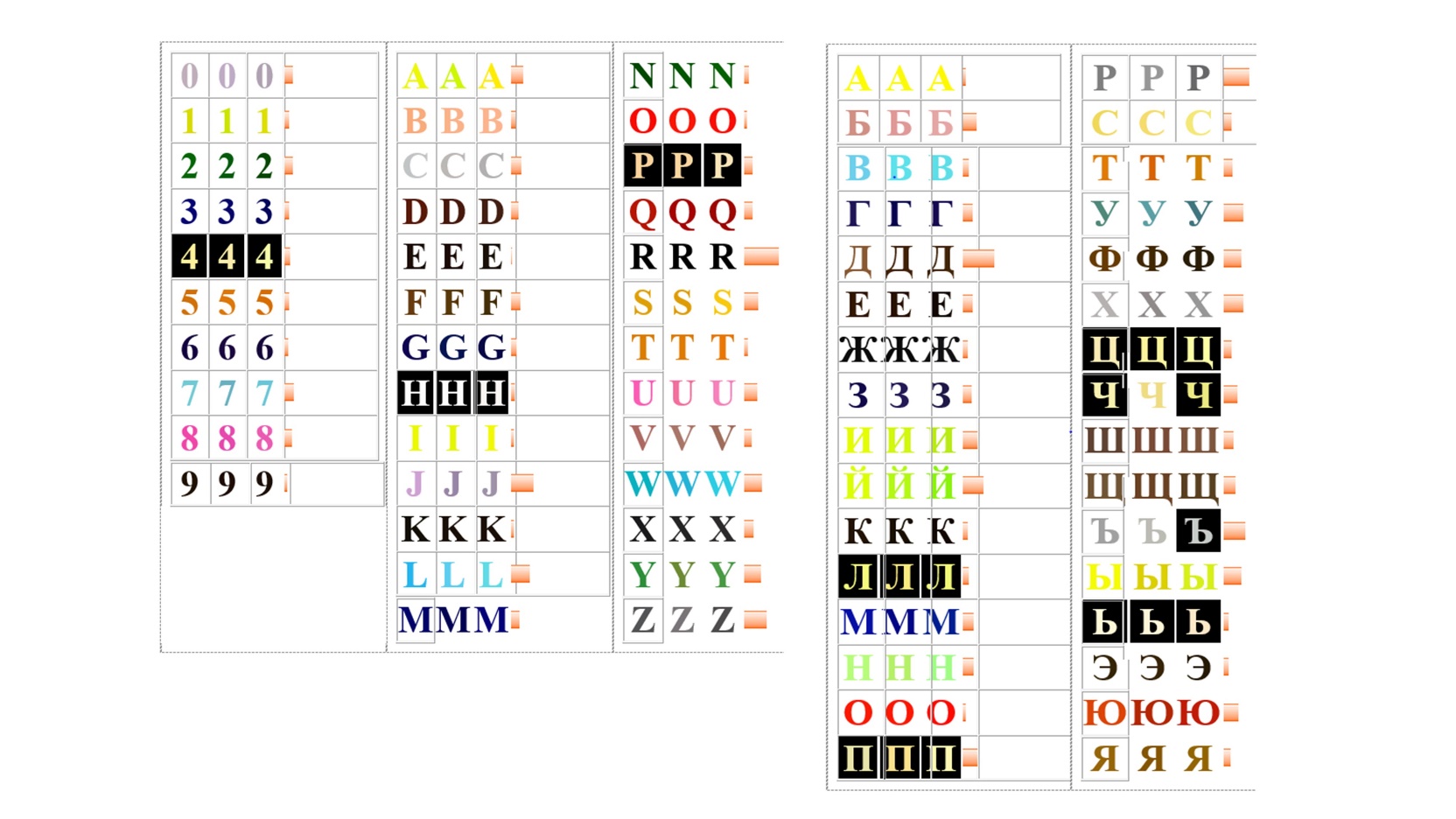


***Supplementary Fig. 2:*** *Results from the synesthesia battery: Latin and Cyrillic alphabet. LP learned the Latin alphabet (German pronunciation) first. The colors of the Cyrillic letters correspond often to the colors of Latin letters with a similar shape (for example the letter M) but if the pronunciation is different, it also affects the color, which is then a mix of shape and sound (example letter H).*

**References**

1. Eagleman DM, Kagan AD, Nelson SS, Sagaram D, Sarma AK. A standardized test battery for the study of synesthesia. J Neurosci Methods. 2007;159(1):139–45.

2. Rothen N, Seth AK, Witzel C, Ward J. Diagnosing synaesthesia with online colour pickers: maximising sensitivity and specificity. J Neurosci Methods. 2013;215(1):156–60.
